# P-1940. Incidence and Outcomes of Pneumocystis carinii Pneumonia in Autoimmune Inflammatory Rheumatic Diseases in Korea: Nationwide study

**DOI:** 10.1093/ofid/ofaf695.2108

**Published:** 2026-01-11

**Authors:** Jin Nam Kim, Kang Ju Son, Wooyong Jeong, Jin Su Park, Bongyoung Kim, Hee Kyoung Choi, Heun Choi

**Affiliations:** Hanyang University College of Medicine, soengdong-gu, Seoul-t'ukpyolsi, Republic of Korea; National Health Insurance Service Ilsan Hospital, Goyang, Kyonggi-do, Republic of Korea; National Health Insurance Service Ilsan Hospital, Goyang, Kyonggi-do, Republic of Korea; National Health Insurance Service Ilsan Hospital, Goyang, Kyonggi-do, Republic of Korea; Department of Internal Medicine, Hanyang University College of Medicine, Seongdong-gu, Seoul-t'ukpyolsi, Republic of Korea; National Health Insurance Service Ilsan Hospital, Goyang, Kyonggi-do, Republic of Korea; National Health Insurance Service Ilsan Hospital, Goyang, Kyonggi-do, Republic of Korea

## Abstract

**Background:**

*Pneumocystis jirovecii* pneumonia (PJP) remains a life-threatening opportunistic infection among immunocompromised individuals, particularly those with autoimmune inflammatory rheumatic diseases (AIIRDs) due to the use of high-dose corticosteroids and other potent immunosuppressive agents. Although prophylactic strategies have been recommended, their effectiveness and risk profiles in AIIRD populations remain unclear. To assess the incidence and outcomes of PJP and evaluate the impact of prophylactic interventions among patients with AIIRDs using a nationwide population-based cohort.

**Table 1.**
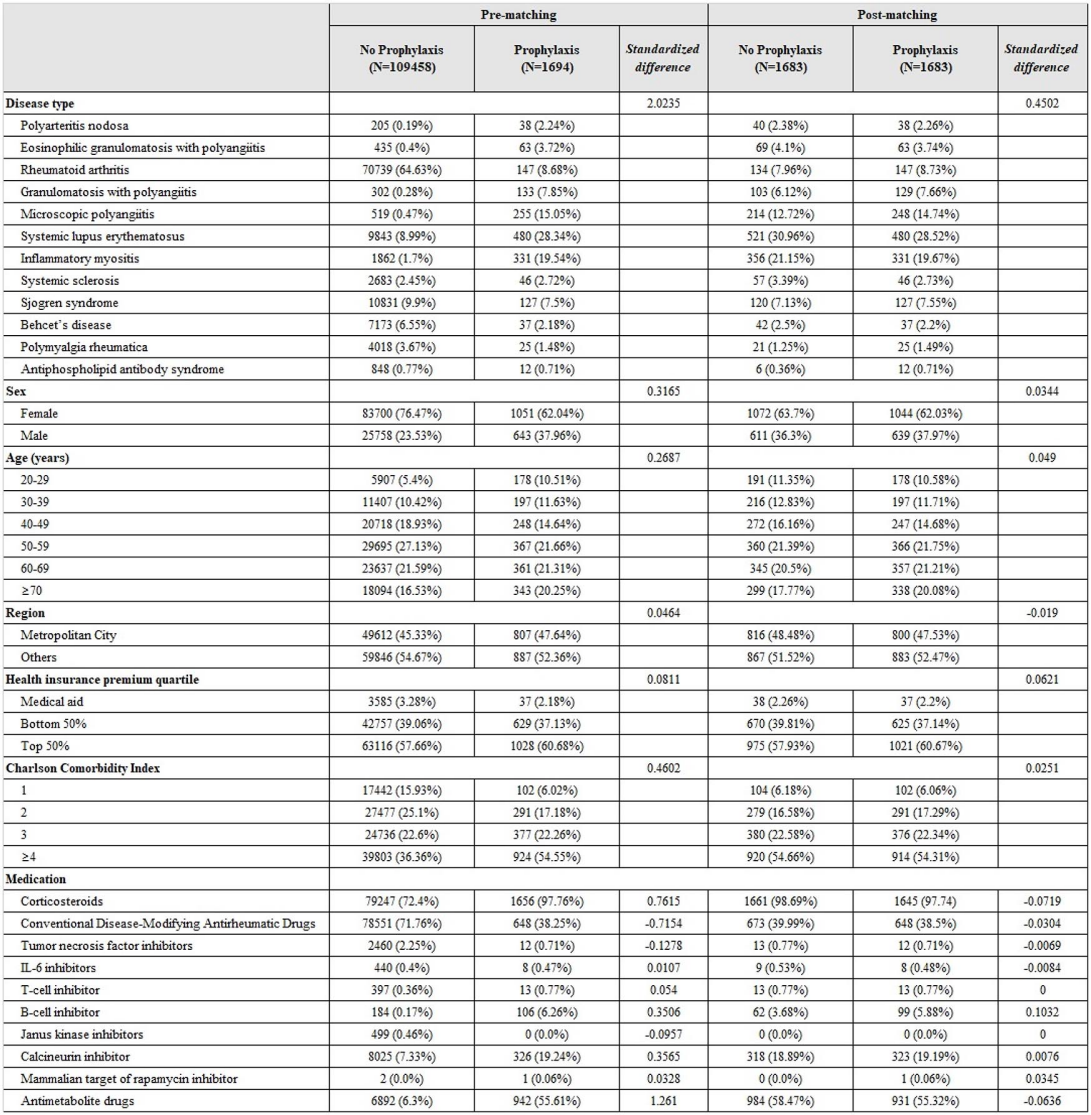
Baseline characteristics of the study population according to prophylaxis before and after propensity score matching

Continuous variables were described as mean and standard deviation (SD), and discrete variables were described as numbers and percentages (%).

**Table 2.**
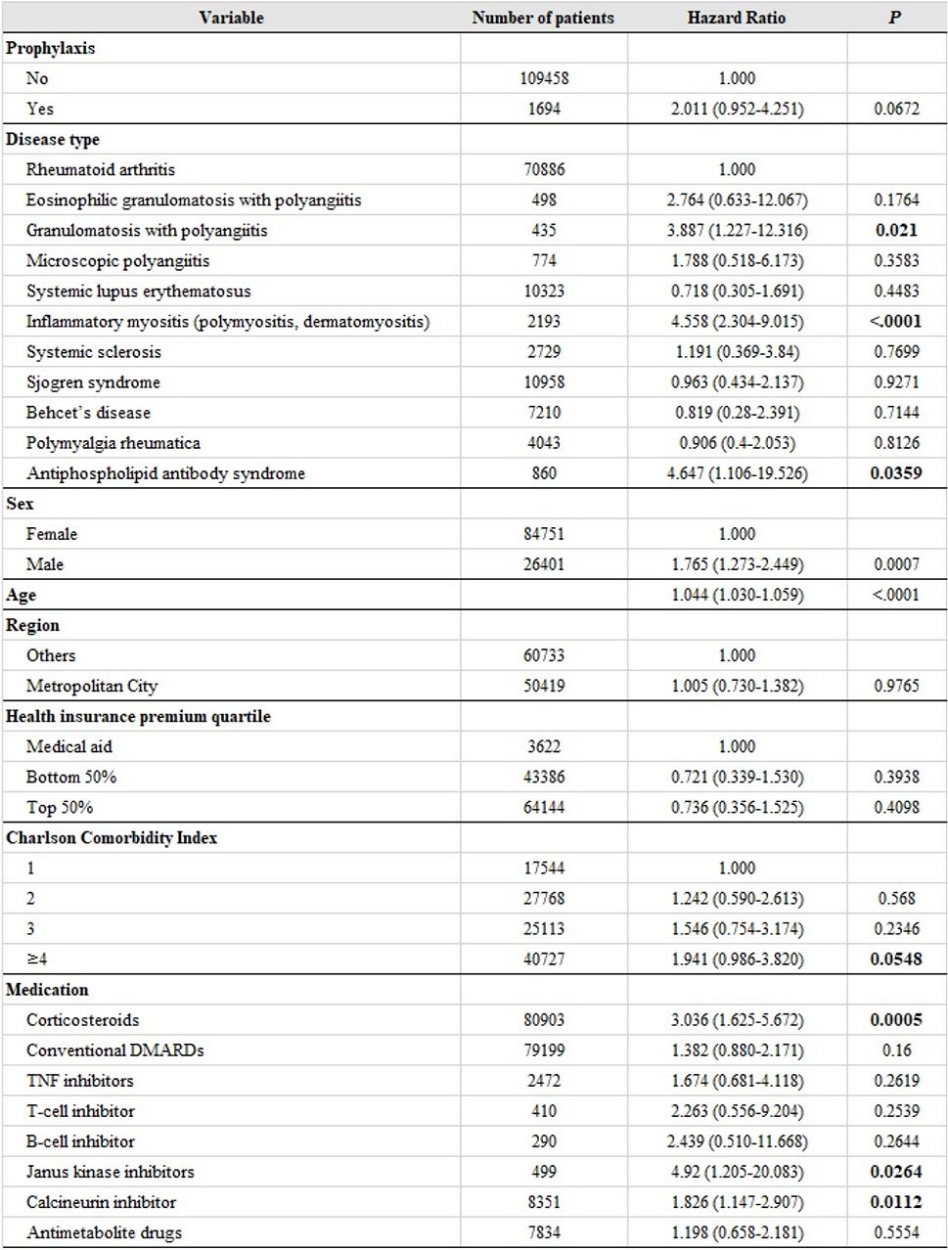
Cox regression analysis of the incidence of Pneumocystis pneumonia in patients with AIIRDs before matching Continuous variables were described as mean and standard deviation (SD), and discrete variables were described as numbers and percentages (%).

**Methods:**

Data were obtained from the Korean National Health Insurance Service database from 2010 to 2022. Patients with AIIRDs were identified using diagnosis and insurance codes and required immunosuppressive agent prescriptions. After applying strict operational definitions and exclusions, 111,152 patients were included. Prophylactic treatment was defined as the prescription of prophylactic agents for ≥90 days within one year of AIIRD diagnosis. Propensity score matching (1:1) resulted in two matched cohorts of 1,683 patients each. The primary outcomes were PJP incidence and all-cause mortality, evaluated using landmark analysis, Kaplan-Meier survival curves, and Cox proportional hazards models.

**Fig 1.**
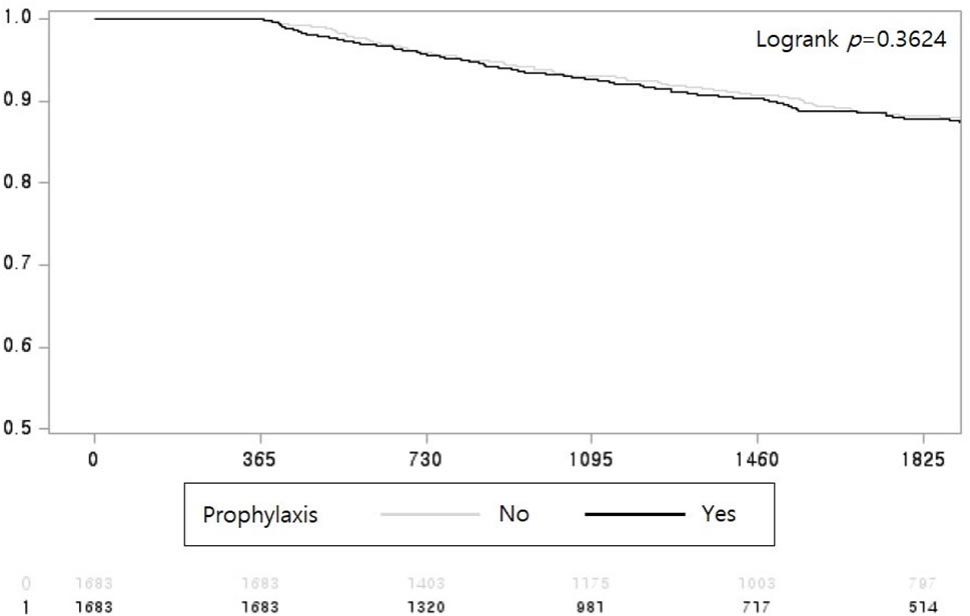
Kaplan-Meier survival curves for all-cause mortality among patients with autoimmune inflammatory rheumatic diseases after propensity score matching

**Results:**

Among the total cohort, 154 patients (0.14%) developed PJP. Risk factors for PJP included granulomatosis with polyangiitis, inflammatory myositis, antiphospholipid antibody syndrome, male sex, older age, corticosteroid use, JAK inhibitor use, and calcineurin inhibitor use. After matching, prophylactic treatment was not significantly associated with reduced PJP risk (HR, 1.586; 95% CI, 0.621–4.049; *P* = 0.3346) and was also not significantly associated with reduced mortality (HR, 1.063; 95% CI, 0.866–1.305; *P* = 0.5611).

**Conclusion:**

PJP incidence in AIIRD patients was low but associated with substantial morbidity. Although prophylactic treatment showed no statistically significant impact on PJP incidence or mortality after matching, specific clinical subgroups exhibited markedly elevated risks. These findings support the need for individualized, risk-adapted prophylaxis strategies among AIIRD patients receiving immunosuppressive therapy.

**Disclosures:**

All Authors: No reported disclosures

